# charmm2gmx: An Automated Method to Port the CHARMM Additive Force Field to GROMACS

**DOI:** 10.1021/acs.jcim.3c00860

**Published:** 2023-07-03

**Authors:** András F. Wacha, Justin A. Lemkul

**Affiliations:** † Institute of Materials and Environmental Chemistry, Research Centre for Natural Sciences, Eötvös Loránd Research Network, Magyar tudósok körútja 2, Budapest H-1117, Hungary; ‡ Department of Biochemistry, Virginia Tech, 111 Engel Hall, 340 West Campus Drive, Blacksburg, Virginia 24061, United States

## Abstract

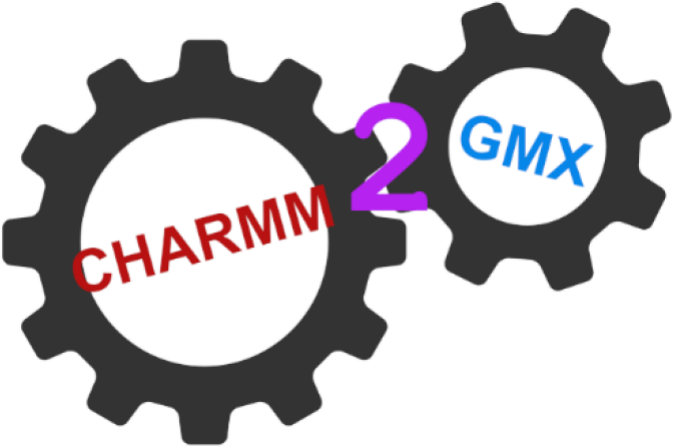

CHARMM is one of the most widely used biomolecular force fields. Although developed in close connection with a dedicated molecular simulation engine of the same name, it is also usable with other codes. GROMACS is a well-established, highly optimized, and multipurpose software for molecular dynamics, versatile enough to accommodate many different force field potential functions and the associated algorithms. Due to conceptional differences related to software design and the large amount of numeric data inherent to residue topologies and parameter sets, conversion from one software format to another is not straightforward. Here, we present an automated and validated means to port the CHARMM force field to a format read by the GROMACS engine, harmonizing the different capabilities of the two codes in a self-documenting and reproducible way with a bare minimum of user interaction required. Being based entirely on the upstream data files, the presented approach does not involve any hard-coded data, in contrast with previous attempts to solve the same problem. The heuristic approach used for perceiving the local internal geometry is directly applicable for analogous transformations of other force fields.

## 1 Introduction

Molecular mechanics (MM) calculations and molecular dynamics (MD) simulations rely on force fields (FFs), namely, the equation used to compute the potential energy of an atomic configuration and all the associated parameters used by this equation. The quality of a FF determines the accuracy of any calculation that uses it. A critically important task among practitioners of simulation methods is ensuring that the employed FF is implemented correctly in the software being used. Different simulation packages offer different benefits – ease of use, simulation speed, available features and algorithms, etc. – therefore presenting users with an important choice, particularly with respect to FF support.

The CHARMM additive FF (henceforth simply CHARMM) is one of the most widely used FFs in biomolecular simulations, covering proteins,^1−4^ nucleic acids,^5−8^ carbohydrates,^9−11^ lipids,^12−14^ and monatomic ions.^15,16^ It has an accompanying general FF, CGenFF, for drug-like molecules.^17^ Thus, the CHARMM FF family covers nearly all entities encountered in biomolecular simulations and has been continually optimized. Given this flexibility, it is desirable to ensure that it is implemented robustly over a wide range of simulation codes. One of the most popular simulation packages in use today is GROMACS,^18,19^ which is free and open-source, and is highly optimized for use on both CPU and GPU hardware.

Implementing CHARMM in GROMACS requires more than just translating measurement units (SI for GROMACS, AKMA for CHARMM); philosophical differences between the two codes must also be harmonized. The CHARMM software has a powerful patching utility that can modify existing residue definitions. In GROMACS, the topology building program pdb2gmx has a limited capacity to generate polymer branch points or modifications to internal chain residues, instead relying almost exclusively on preconstructed residue definitions. This second challenge is considerably more difficult, though critical for the use of the CHARMM FF, in which many modified amino acids, nucleotides, etc., are not present as residues, rather as patches that the user applies in the script-based CHARMM interface.

CHARMM is organized in a modular fashion. The core is specified in pairs of parameter (*.prm) and topology (*.rtf) files. Additional residue definitions and associated parameters are available in “stream” files (*.str), which often contain both topology and parameter information. Typically, the user selects and imports only the relevant residues and parameters needed to describe their system in their CHARMM script. Some naming conflicts may arise, particularly when the “core” CHARMM FF is used in concert with CGenFF, as model compounds may have the same names. This situation is a natural outcome of needing a large set of compounds in CGenFF so new ones may be parametrized by using them as reference. Therefore, it is the responsibility of the user to select which version of these molecules they wish to use. In contrast, FFs are monolithic in GROMACS, and thus these redundancies need to be resolved at the time the port is made. As such, some decisions need to be made as to which residue definitions to retain, and while a “canonical” reference port is distributed, it cannot be expected to fit all needs.

Several programs have been written to address the challenge of implementing the CHARMM FF in GROMACS, many resulting from individual investigators’ needs to have access to certain species. These scripts and programs have been passed around privately among different practitioners and also shared on the GROMACS “User Contributions” Web site over many years. Though useful, these utilities rarely provided any evidence of robustness.

The first documented attempt at porting CHARMM under GROMACS, i.e., expressing the data in a format readable by the latter, was the implementation of CHARMM22/CMAP (colloquially referred to as “CHARMM27”).^20^ In doing so, the authors extended the GROMACS engine itself to enable treatment of Urey–Bradley angle potentials, multiple dihedrals with different multiplicities, and dihedral crossterm energy correction maps (CMAP). While much of the work, such as the conversion of interaction parameters and basic residue topology data, was done in an automated way, a substantial amount of data was hardcoded, rather than analyzing and converting the corresponding parts of the original FF files. Examples include some residue topologies originally defined in CHARMM as “patches,” as well as most of the required input files of the pdb2gmx tool (termini, hydrogen addition database, virtual site database, etc.). The obvious drawback, apart from the tedium of manual work and the possibility of errors, is the problem of maintenance. That is, changes introduced upstream are not necessarily recognized or otherwise require continual, manual intervention by those maintaining the FF port.

More recently, the CHARMM-GUI web server has emerged as an important tool in producing GROMACS inputs for systems using the CHARMM FF.^21^ Internally, CHARMM-GUI will convert a PSF topology to the GROMACS format and will write out the required subset of the CHARMM FF needed by GROMACS. This approach is efficient for the end user, who navigates a series of steps to produce everything needed to run the simulation. The drawback is that the user is not provided with the entire FF, and therefore, if changes are made to the prepared system (e.g., introduction of a ligand, mutation or modification of a residue, etc.), the files may no longer be suitable even if the topology can be regenerated.

Based on these previous efforts, we created charmm2gmx, a generalizable, fully automatic conversion utility for FF porting. We sought to minimize hardcoding, using heuristics and depending strongly on the upstream data. Required user input is aggregated in a single input file for the sake of repeatability and self-documentation. Another important requirement was to remain as close as possible to the conventions of nomenclature, data organization, and logic of the original FF release. Finally, in the sense of Linus’s law, i.e., “given enough eyeballs, all bugs are shallow,”^22^ we make our conversion script freely available under a permissive license.

## 2 Implementation Details

The charmm2gmx conversion utility is written in Python, the *de facto* general purpose programming language of scientific computing with a large user base.^23,24^ As an interpreted language, its performance is nowhere near that of compiled languages like C/C++ or Fortran, but the gain in development effort and code maintainability more than compensates for this tradeoff.

### 2.1 Reproducibility

The conversion process is controlled by a single input file, conventionally named charmm2gmx.in (an example is given in the Supporting Information). Its main role is to specify the topology, parameter, and stream files to be converted, being similar in this sense to the beginning part of an input file for the CHARMM interpreter. It is also in this file where the conversion process can be tailored. By distributing the input file with a port of the FF, reproducibility can be ensured.

### 2.2 Bonded and Nonbonded Parameter Conversion

Bjelkmar et al. implemented the CHARMM additive FF functional form in GROMACS and showed that the difference in potential energy surface obtained by the two engines is negligible.^20^ Parameter conversion is mostly straightforward, requiring only unit conversion and a factor of 2 in harmonic potential force constants. The resulting values are all written to the bonded parameter file (ffbonded.itp).

The only nontrivial part here is the case of the Lennard-Jones (LJ) interaction parameters. CHARMM defines the *ε* and *R*_min_/2 parameters for each atom type, from which the appropriate parameters for each pair of atom types are derived automatically using the Lorentz–Berthelot combining rules. As is typical for biomolecular FFs, LJ interactions are excluded between first- and second-neighbor atoms, while third-neighbor interactions (also called 1–4 interactions) are scaled down by some factor. Instead of scaling, CHARMM introduces a second set of LJ parameters for some 1–4 interactions, which, when present, override the standard LJ parameters. The corresponding topological directive in GROMACS is “pairs,” and a direct enumeration of 1–4 LJ interaction parameters is required under the “pairtypes” directive in the FF file corresponding to all nonbonded interactions (ffnonbonded.itp). Similarly, CHARMM defines specific combinations of LJ parameters between nonbonded pairs that can override values arising from combination rules. These so-called NBFIX terms are detected in CHARMM parameter files and added under “nonbond_params” to a GROMACS-formatted topology file called nbfix.itp.

### 2.3 Residue Topology Conversion

Residues are the building blocks of the molecular topology, either as standalone entities or as constituents of macromolecules. CHARMM organizes residues in groups, e.g., “prot,” “carb,” “na,” “lipid,” and “cgenff” for proteins, carbohydrates, nucleic acids, lipids, and the small molecules comprising the core of the CGenFF, respectively. In charmm2gmx, this organization can be preserved by writing the separate residue topology files in GROMACS format (extension *.rtp). This organization facilitates the implementation of CHARMM in GROMACS by maintaining the nomenclature that is familiar to CHARMM users.

Atom properties (name, type, and partial charge) are transferred directly. Although the Verlet cutoff scheme has superseded the now obsolete group-based method for treating nonbonded interactions in recent versions of GROMACS, atom grouping into integer charge groups is preserved. We feel that this helps the user to better understand the topology and organization of the residue. Doing so makes direct comparisons of the CHARMM and GROMACS files easier and enables users to learn the functionalities and conventions of each program. We note that these group assignments are ignored by GROMACS utilities grompp and mdrun for compiling a run input file and performing simulations.

Atom connectivity in CHARMM (bonds, improper dihedrals, and CMAP terms) is also readily translated. The occasional bond order specification (double or triple) is discarded, as no such distinction is made in the GROMACS residue definitions. Thus, all bond declarations are treated the same. Hydrogen bond donors and acceptor specifications, used only by CHARMM analysis routines, have no equivalent in GROMACS and are discarded.

CHARMM residue definitions also contain internal coordinate (IC) tables that define either an optimized geometry of the residue or dummy values that can be populated from bond and angle values taken from the parameter file. The IC tables allow users to build any molecule entirely from scratch or to reconstruct any atoms that are missing from the original structure. As this information is optional and GROMACS does not have an equivalent function (except for the specific case of building missing hydrogen atoms), the IC tables are also discarded. A direct comparison between CHARMM and GROMACS residue definitions for alanine is shown in Figure S1.

### 2.4 Heuristic Perception of Local Geometry

Although the topology building utility of GROMACS (pdb2gmx) has only a limited capacity to construct new atoms compared to the full flexibility of CHARMM’s IC builder, it can still perform a useful subset of topology and coordinate operations. The required information is not directly available in CHARMM but can be derived using a heuristic approach we implemented in charmm2gmx.

When the topology of the system is constructed, both the CHARMM interpreter and pdb2gmx generate entries based on bonded connectivity information to generate angles and proper dihedrals. We do the same after converting the residue topologies to the GROMACS format: by enumerating three- and four-atom interactions and assigning actual numeric parameters based on the types of the participating atoms, the internal geometry of the molecule can be approximated. As described below, this information turns out to be useful in sanity-checking the ported FF and constructing atom addition rules. Although this heuristic method for perceiving the local geometry is less precise, it is still applicable, even when IC data are incomplete or absent.

The core of the algorithm is the concept of the “bonded tree,” branching out from a root atom through bonds declared in the residue topology and avoiding rings. Enumeration of valence angles is achieved by constructing the bonded trees up to the second neighbor for all of the atoms in the residue. Proper dihedral terms are obtained in a similar fashion, but with a maximum depth of 3 bonds. Repeated entries (e.g., A-B-C vs C-B-A) are discarded.

In the case of chainable residues (amino acids, nucleotides, etc.), an infinite homopolymer of the same building block is assumed. In the case of terminal residues (e.g., acetyl or *N*-methylamide termini), linking to the appropriate end of a semi-infinite homopolymer of all possible internal residues is attempted.

### 2.5 Topology Patching

Patching is a very powerful capability of CHARMM, whereby both the topology and the corresponding coordinate set can be altered by adding, modifying, or removing atoms and bonded interactions. It is used for several different purposes, effectively expanding the number of available residues without introducing redundant residue definitions by relying on PRES (“patch residue”) entries that can be applied to one or more existing residues. The PRES entries have the same general format as ordinary RESI (residue) entries but differ in that atoms/interactions can also be deleted, and the specified atoms and interactions are additions/replacements without distinction. Below, we describe how a subset of this powerful aspect of CHARMM can still be implemented in GROMACS.

#### Patched Residues

Several residue topologies in CHARMM are implemented as minor modifications to a base residue (protonation states, phosphorylation, etc.). Deoxyribonucleotides are also derived from the corresponding ribonucleotides. Because GROMACS topology generation by pdb2gmx requires that all residues be defined in *.rtp files, previous efforts to include patched residues relied on manually generating the altered residues. We improved upon that approach by allowing patches to be mapped onto base residues to generate new GROMACS-compatible residue definitions. As the atom additions, modifications, and deletions are clearly specified in the CHARMM residue topology file as well as all new bond or improper dihedral terms, we automated the topology patching process in charmm2gmx. For each such case, the names of the base residue, the patch, and the final patched entry can be given in the charmm2gmx.in input file, and the conversion utility takes care of the rest.

#### Termini Database

Some patch entries are used for specifying modifications to residues to generate chain-terminating versions, such as those that occur at the N- and C-termini of polypeptide chains or the 5′- and 3′-termini of oligonucleotides. When pdb2gmx recognizes a contiguous chain in the input coordinate file, the starting and ending residues are identified and can be modified either automatically or by user choice. Information on the applicable modifications is stored in the termini database files *.n.tdb and *.c.tdb for N- (or 5′-) and C- (or 3′-) termini, respectively. Constructing entries in these databases is not straightforward because there are modifications that occur to the molecular geometry (addition or deletion of atoms) and/or the topology (modification of charges and/or atom types). To provide automatic support for this conversion, a set of atoms must be identified that constitutes the minimum for defining the geometry and must then be unified with an internal chain residue for comparing and modifying the topology. In addition to atom addition, deletion, and modification in the CHARMM engine, GROMACS supports atom renaming, which presents an additional challenge for the conversion.

Our approach separates the two conceptionally different tasks of modifying the molecular geometry and adapting an internal residue into a terminal one. Atoms with different names are considered different even if they occupy the same position in the molecule. The rules for creating a terminus entry are as follows:

1. Atoms that are explicitly removed by the current terminus from the base residue are deleted using [delete] directives
2. Atoms in the base residue that are modified by the current terminus:
  a. If the name changed, add a [delete] directive and list them in an [add] entry. This approach resembles the CHARMM philosophy.
  b. If only the type/partial charge changed, add a [replace] directive
3. Atoms added by the current terminus to the base residue: [add] directives are generated. If multiple atoms of different type or partial charge are added with the same directive, generate [replace] directives when needed.
4. Atoms present in other termini but not in the current one: add [delete] directives
5. Add [delete] directives for frequently appearing terminal atoms (e.g., OXT) that do not belong to any known terminus entry. The list of these atoms can be given in charmm2gmx.in.

**Figure 1:**
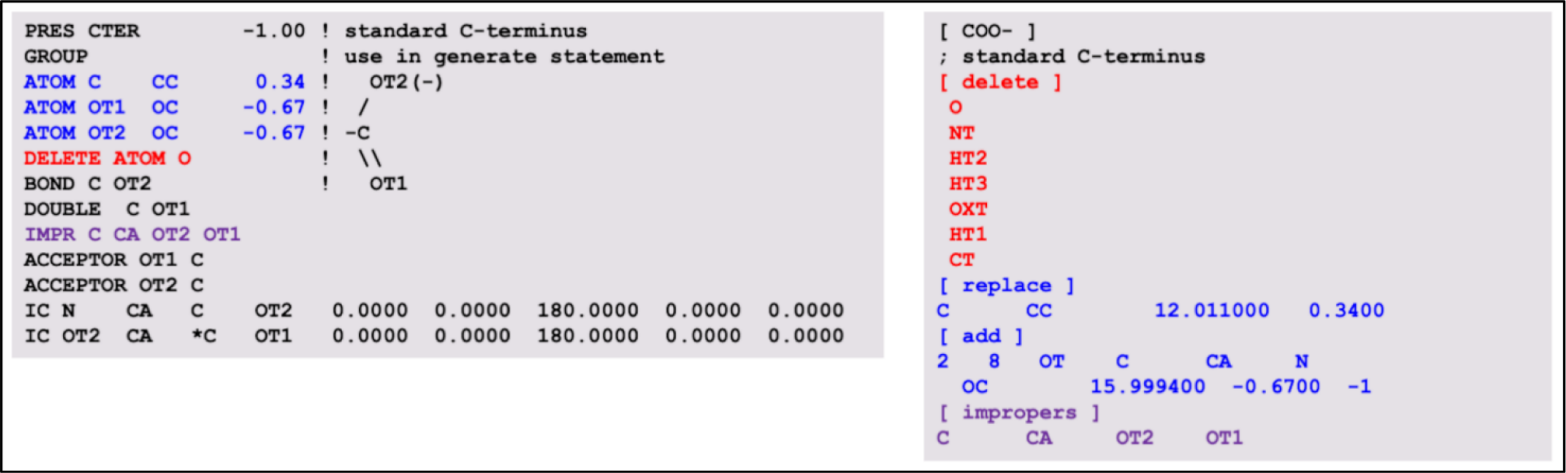
Comparison of CHARMM PRES entry (left) and GROMACS *.tdb (right) formats. Equivalent content is shown with matching colors. BOND and DOUBLE information that is explicitly shown in the CHARMM entry is implied in the GROMACS entry and is handled internally by the pdb2gmx code. DONOR, ACCEPTOR, and IC information is discarded.

The process described above is easier to automate (as shown in the example of carboxylate C-terminus in Figure 1), at the cost of inducing minimal changes in the positions of some atoms by deleting them first and adding them back later. However, the introduced residual strains are easily resolved by a short energy minimization afterward.

#### Cross-Linking

Rules for introducing branch points in a linear polymer are written in specbond.dat, a file located in the top-level data directory of the GROMACS software distribution. This file is FF independent and is the definitive source of the “canonical” names of the cross-linked variants of various molecules and residues. The patches used by CHARMM for this case modify two residues simultaneously. Therefore, residue definitions for cross-linked residues can be generated for use in GROMACS by applying half of the corresponding patch to the appropriate base residue topology, as described above.

### 2.6 Hydrogen Addition Rules

Missing hydrogen atoms can be built by pdb2gmx if the required definitions of internal geometries are present in hydrogen addition database files (*.hdb in the FF directory). The placement of the hydrogens is governed by the covalently bonded heavy atom and two or three other so-called control atoms, which are dependent upon addition rules that map to internal geometry specifications, enumerated in the GROMACS Reference Manual.^25^

Based on the geometry perception heuristics described above, hydrogen addition rules can be generated for almost all residues, an operation that should ultimately be agnostic of a specific FF. Based on the perceived local geometry (including angles and dihedrals) and the chemical type of the heavy atom, the addition rule and control atoms are chosen. Planarity is detected by the existence of an improper dihedral term with a 0° or 180° minimum energy position. Proper symmetry (i.e., the defined multiplicities) typically reflects the rotational symmetry around the bond, making them also suitable for detecting planarity or 3-fold rotational symmetry. Tetrahedral or planar configurations are also checked by looking at the energy minima of the bond angle terms. We found that this approach succeeds in producing hydrogen database entries for almost all residues. Some results are admittedly approximate; however, a short energy minimization of the initial coordinates should be able to fix these cases, too.

### 2.7 Auxiliary Data

#### Water Models

FFs in GROMACS traditionally supply several water models. Since these are not frequently changing and do not strictly belong to the FF itself, we implemented the water models in the porting code itself. Currently, the original^26^ and the CHARMM-modified TIP3P,^27,28^ SPC,^29,30^ SPC/E,^31^ TIP4P,^26^ TIP4P-Ew,^32^ and the TIP5P^33^ water models are supported. These models are made available for the cases in which users wish to compare simulation outcomes with a different model, but since CHARMM is internally calibrated against the CHARMM-modified TIP3P via individual water interactions in partial charge assignment and validation, it is considered tightly linked to the FF.

#### Citation Handling

References to relevant FF publications are interspersed throughout the CHARMM FF files as comments. Automatically detecting them is impossible, but communicating these references to users is important so that proper citations can be made and users have facile access to critical information about the parametrization and validation of entities they wish to use. To solve this challenge, charmm2gmx accepts a BibTeX-formatted citation database file, so that citations can be included in the forcefield.doc file.

### 2.8 Other Quirks

Some features of the CHARMM engine are either implemented differently or not at all in GROMACS. In order to make the produced FF usable, some workarounds have to be made.

#### Lone Pairs

Some residues, e.g., chlorobenzene (code CHLB), use virtual sites to mimic the σ-hole of the halogen atom.^34^ While GROMACS supports the construction of these lone pairs as virtual sites, pdb2gmx cannot generate the required topology directives. Such residues are therefore discarded during conversion. Should virtual site writing be added to pdb2gmx, then the inclusion of these residue definitions would be simple to implement.

#### Missing Parameters

In two special cases, CHARMM and GROMACS behave differently when assigning numeric values of the interaction parameters to bonded interaction entries in the topology. The first one is collinearity in proper dihedrals: whenever one or two neighboring valence angles are 180°, the dihedral angle is undefined. In these cases (e.g., 2-butyne, residue 2BTY in CGenFF), the corresponding dihedral parameters are not even defined in the CHARMM parameter file. The CHARMM program recognizes collinearity by checking the IC tables and avoids generating the dihedral term; therefore, the “missing” parameter does not cause an error. The pdb2gmx utility cannot perform this check and will generate all possible dihedrals. Later, the grompp command will complain about missing dihedral parameters. This situation is remedied by a separate subprogram of charmm2gmx (details in the Supporting Information) which, when called after the conversion process, can detect these “missing” dihedrals and add “dummy” dihedral types with zero force constant, having no impact on the forces but overcoming an error that is not actually a problem the user needs to solve.

Improper dihedrals may also cause problems related to the order in which the atoms are defined in the interaction. S-adenosyl-homocysteine (residue SAH in CGenFF) defines two improper dihedrals in the adenine moiety for which parameters do not exist for the types of atoms in the *i-k-j-l* sequence but do for *i-k-j-l* in one case and *i-l-k-j* in the other. CHARMM allows such permutations, since the order of the defined atoms around the central atom is arbitrary. GROMACS allows only the *l-k-j-i* form. This situation is also recognized by the above-mentioned subprogram and resolved by replicating the improper dihedral types with the permutation of atoms that a residue topology was found to require. These additional parameters are written into ffmissingdihedrals.itp instead of ffbonded.itp for clarity.

#### Deviations from CHARMM Nomenclature

The current CHARMM port adopts some GROMACS naming conventions for termini (e.g., NH3+ in GROMACS vs NTER in CHARMM) but supplies acetylated N-terminus (ACE) and *N*-methylamide C-terminus (NME) as standalone residues instead of termini. The latter is necessary because pdb2gmx does not have the appropriate atom addition rules to modify the ends of the peptide sequence.

In addition to the above, the naming of atoms in ACE and NME has been different from the CHARMM nomenclature (e.g., CH3 instead of CAT or CAY) in the previous ports, following the conventions of the OPLS/AA and AMBER force fields. Such changes to the convention also interface more smoothly with default atom group definitions in GROMACS, such as which atoms are considered part of the backbone for the purposes of RMSD calculations and other operations.

A third point of difference concerns the name of ions, e.g., SOD vs NA and CLA vs CL in CHARMM and GROMACS, respectively. Notably, the former names are used in GROMACS topologies produced by CHARMM-GUI, while the latter corresponds to the traditional GROMACS terminology. As a convenience to the users, both versions can be generated by the conversion script using the “copyresidues” keyword (see the Supporting Information for more information).

## 3 Validation

Following the conversion of an FF from one format to another, it is essential to determine if the two implementations produce identical forces for equivalent atomic configurations. If the two programs agree in their results, users can expect equivalent results from the two programs, assuming no other relevant bugs are present in the code (ascertaining that is beyond the scope of this work). Here, we validated energies produced by the CHARMM port across a subset of residues, covering many different chemical types and thus capturing a broad cross-section of the CHARMM FF. For each residue tested, we obtained the potential energy of the system via a single-point energy evaluation. As it is practically impossible to reproduce identical trajectories between different simulation engines given issues related to nondeterministic seeds in various algorithms, limitations of floating-point rounding, order of operations, etc., the most robust validation that can be done is a detailed analysis of the potential energy terms and forces within identical configurations of atoms. We used the potential energy only as a proxy for the forces in the system since they are directly linked.

Amino acids were tested in the context of two sets of tripeptides, Ala-Xaa-Ala and (Xaa)_3_, where Xaa is the amino acid of interest and DNA and RNA nucleotides were built as trinucleotides with the same base. Doing so allows for a complete assessment of terminal and internal residues, thus validating patches for terminal residues at the same time. All 20 canonical amino acids were included in the testing, as were all five canonical nucleobases. Alanine dipeptide was generated as a special case for the protein FF, to evaluate the validity of acetyl and *N*-methylacetamide capping groups; otherwise, the Ala-Xaa-Ala tripeptides were constructed with ionized termini to model typical usage cases, and the (Xaa)_3_ tripeptides use neutral termini to account for terminus-specific cases. Coordinates were initially generated in CHARMM via its IC builder, and any atomic nomenclature differences (see above) were handled by a Bash script that replaces atom names with standard Linux command-line utilities like sed. Methodological details of the energy calculations and enumerated results are provided in the Supporting Information, Tables S1–S9.

Overall, the single-point energies are reasonably reproduced between each software package (on the order of 0.01 kcal mol^–1^ or less), indicating that the FF port generated by charmm2gmx is robust. The largest discrepancies occur in the condensed-phase systems, which systematically differ by ∼4–5 kcal mol^–1^ in the LJ term (Tables S8 and S9), caused by the different handling of neighbor lists (recent versions of GROMACS support only buffered Verlet lists). This small deviation does not result in forces that differ substantially on a per-atom basis (the difference is only ∼0.1% of the LJ term), and both packages have been shown to produce equivalent results.^21^

To illustrate the correctness of the ported FF, we performed actual simulations with the CHARMM FF using different MD codes on two systems: an aqueous solution of lysozyme and a lipid bilayer patch containing 64–64 dipalmitoylphosphatidylcholine (DPPC) molecules in each leaflet. Input files for GROMACS, CHARMM and OpenMM were generated using the Solution Builder and Membrane Builder tools of CHARMM-GUI.^21,35,36^ Another simulation has been based on the GROMACS input files, where the topology of the system has been rebuilt using the pdb2gmx utility of GROMACS, employing the FF port made with charmm2gmx, but otherwise following the same equilibration procedure. Commonly analyzed quantities, such as the root-mean-square deviation of a reference structure, radius of gyration of the protein, area per lipid, membrane thickness average angle adopted by the lipid tails with the membrane normal, and the electron density along the bilayer normal, were derived from the trajectories and compared. All above quantities were found to closely match, regardless of the MD engine and the FF port used (Figures S2–S7 in the Supporting Information).

## 4 Conclusions

We have presented charmm2gmx, a utility written in the Python language to automatically convert CHARMM-formatted topology and parameter files into GROMACS format. Our goal was to produce a robust code that facilitates the use of a popular nonpolarizable FF in widely used GROMACS software. To demonstrate the validity of this software, we performed extensive single-point energy analysis and MD simulations on a variety of molecular systems. We have demonstrated that the facile conversion of FF files yields a reliable port of the CHARMM FF into the GROMACS format, thereby allowing broad use by the simulation community. Although developed primarily with the CHARMM FF in mind, charmm2gmx can serve as the basis for conversion methods for other FFs. Notably, the heuristic approach for perceiving local internal geometry should directly be usable to construct hydrogen addition rules and perform sanity checks in the GROMACS ports of other FFs.

## Data Availability

The charmm2gmx code and the data and scripts used for validation are available free of charge at https://awacha.gitlab.io/charmm2gmx/index.html. The CHARMM force field files can be downloaded from Alexander MacKerell Jr.’s homepage (http://mackerell.umaryland.edu/charmm_ff.shtml), where the “canonical” GROMACS ports made by the present software are also available. Data and scripts used for validation with MD simulations are deposited on Zenodo (https://doi.org/10.5281/zenodo.7997865).

## Supporting Information Available

Text describing file formats and usage of charmm2gmx subcommands, along with a detailed description of the methods used for validating energies, tables listing the potential energies of tested molecules in both CHARMM and GROMACS, figures illustrating topology equivalences and structural properties from the validation MD simulations, and sample charmm2gmx input file (PDF)
Potential energy terms of the tested molecules (XLSX)

## Abbreviations

MD: molecular dynamics
PME: particle mesh Ewald
LJ: Lennard-Jones
IC: internal coordinates
amu: atomic mass units
DPPC: 1,2-dipalmitoyl-*sn*-glycero-3-phosphatidylcholine
