# Supplementary material for: charmm2gmx: An Automated Method to Port the CHARMM Additive Force Field to GROMACS
Source: J Chem Inf Model. 2023 Jul 3;63(14):4246–52. doi: 10.1021/acs.jcim.3c00860 (PMC10369483; doi:10.1021/acs.jcim.3c00860)
Supplement: Supplementary file 1 — ci3c00860_si_001.pdf [file ci3c00860_si_001.pdf]

Supporting Information

charmm2gmx: An Automated Method to Port the CHARMM Additive Force Field to GROMACS

András F. Wacha

Institute of Materials and Environmental Chemistry, Research Centre for Natural Sciences,

Eötvös Loránd Research Network, Magyar tudósok körútja 2, Budapest, H-1117, Hungary

wacha.andras@ttk.hu

Justin A. Lemkul

Virginia Tech Department of Biochemistry, 111 Engel Hall, 340 West Campus Dr. Blacksburg,

VA 24061, United States of America

Comparison of CHARMM and GROMACS residue topology definitions

```
RESI ALA          0.00
GROUP
ATOM N    NH1    -0.47  !     |
ATOM HN   H       0.31  !  HN-N
ATOM CA   CT1     0.07  !     |     HB1
ATOM HA   HB1     0.09  !     |    /
GROUP                   !  HA-CA--CB-HB2
ATOM CB   CT3    -0.27  !     |    \
ATOM HB1  HA3     0.09  !     |     HB3
ATOM HB2  HA3     0.09  !   O=C
ATOM HB3  HA3     0.09  !     |
GROUP                   !
ATOM C    C       0.51
ATOM O    O      -0.51
BOND CB CA  N  HN  N  CA
BOND C  CA  C  +N  CA HA  CB HB1  CB HB2  CB HB3
DOUBLE O  C
IMPR N -C CA HN  C CA +N O
CMAP -C  N  CA  C   N  CA  C  +N
DONOR HN N
ACCEPTOR O C
IC -C   CA   *N   HN    1.3551 126.4900  180.0000 115.4200  0.9996
IC -C   N    CA   C     1.3551 126.4900  180.0000 114.4400  1.5390
IC N    CA   C    +N    1.4592 114.4400  180.0000 116.8400  1.3558
IC +N   CA   *C   O     1.3558 116.8400  180.0000 122.5200  1.2297
IC CA   C    +N   +CA   1.5390 116.8400  180.0000 126.7700  1.4613
IC N    C    *CA  CB    1.4592 114.4400  123.2300 111.0900  1.5461
IC N    C    *CA  HA    1.4592 114.4400 -120.4500 106.3900  1.0840
IC C    CA   CB   HB1   1.5390 111.0900  177.2500 109.6000  1.1109
IC HB1  CA   *CB  HB2   1.1109 109.6000  119.1300 111.0500  1.1119
IC HB1  CA   *CB  HB3   1.1109 109.6000 -119.5800 111.6100  1.1114
```

```
[ ALA ]
;
 [ atoms ]
      N     NH1 -0.4700   1
     HN       H  0.3100   1
     CA     CT1  0.0700   1
     HA     HB1  0.0900   1
     CB     CT3 -0.2700   2
    HB1     HA3  0.0900   2
    HB2     HA3  0.0900   2
    HB3     HA3  0.0900   2
      C       C  0.5100   3
      O       O -0.5100   3
 [ bonds ]
     CB    CA
      N    HN
      N    CA
      C    CA
      C    +N
     CA    HA
     CB   HB1
     CB   HB2
     CB   HB3
      O     C
 [ impropers ]
      N    -C    CA    HN
      C    CA    +N     O
 [ cmap ]
     -C     N    CA     C    +N
```

Figure S1. Comparison of CHARMM RTF (left) and GROMACS RTP (right) formats. Equivalent information is shown in matching colors. DONOR, ACCEPTOR, and IC lines in the CHARMM RTF entry are ignored in the GROMACS implementation.

CHARMM2GMX subcommands

Similar to the execution of all GROMACS programs as subsidiary modules of the `gmx` command, all functionality of the force field conversion utility is available through subcommands of the `charmm2gmx` executable. Below, we list the subcommands and summarize their functions.

convert. The main functionality of `charmm2gmx` is converting the force field data from CHARMM to GROMACS format. This process is performed by the `convert` subcommand,

which accepts a single argument, the location of a plaint-text input file (usually `charmm2gmx.in`). For the sake of self-documentation, all characters on the line including and after a semicolon ("`;`") are treated as comments and disregarded by the processor, in the same way the comments are specified in GROMACS files.

Contents of the input file are organized in sections, corresponding to molecule classes in the CHARMM force field (e.g. "prot" for proteins, "na" for nucleic acids, "carb" for carbohydrates, etc.). Section headers are given in square brackets, as in almost all GROMACS input files. Topology data written by the conversion utility (i.e. `*.rtp`, `*.n.tdb`, `*.c.tdb`, and `*.hdb` files) will be prefixed by the section name. Other lines of the file have the format "`directive: arguments`". Some directives require an option, which can be given in brackets as "`directive[option]: arguments`". A simplified scheme of an input file is shown in Figure 3, while a more complete one, heavily documented and used for preparing the "official" 2022 port can be found in the Supporting Information.

On the top level of the file, before the first section is declared, five directives control the operation of the conversion utility: "`charmmversion`" and "`cgenffversion`" declare the version of the parent CHARMM force field and the associated CGenFF. Input and output directories are given by "`toppardir`" and "`outputdir`," respectively. Additionally, a BibTeX-formatted citation database can be given with the "`bibtexfile`" directive, to include references in the output.

Force field data files are defined under each section with the "`prm`," "`rtf`," or "`str`" directives, followed by the file system path of the actual input file, relative to the top directory declared with the "`toppar`" top-level directive. `charmm2gmx` reads and parses the files in the order they appear in the input file.

Due to the modularity of the CHARMM force field, multiple, conflicting definitions of interaction parameters and residue topologies may exist in different files. When encountering such a situation, the conversion utility stops with an error message. To remedy this situation, each file declaration directive can optionally be prefixed with either a + or – character, controlling the precedence of the data in that file over the others. All interaction parameters and residue topologies which appear in a file declared with a '+' line prefix will override other declarations. On the other hand, declaring a file on a line starting with '–' will allow all parameters and topologies be overridden, if redefined elsewhere.

Behavior of the conversion utility can be further tailored in each section. A whitespace-separated list of residue names can be specified with the "`overrideresidues`" or "`discardresidues`" directives for a more fine-grained control of precedence. Residue topologies can be renamed or copied using "`renameresidues`" or "`copyresidues`" directives. With "`renameatoms[residuename]`" atom names can be modified for compatibility with the established GROMACS nomenclature.

For the resolution of parameter conflicts, the "`renameatomtype`" directive is useful. It replaces all occurrence of the specified atom type with a new name, including in the most recently read input file. It may be required to repeat this directive for files depending on each other.

Handling of the directives related to patching is postponed until after all input files have been read and parsed. This timing means that they can cross over section barriers to use information from all sections. Using the "`patch`" directive, a patch residue topology can be applied onto a base, resulting in a stand-alone residue topology entry. Directives "`nter`" and "`cter`" convert the named residues to entries in the termini database. A base residue topology needs to be given in brackets. Finally, with "`nter_delete`" and "`cter_delete`" directives, one can specify

atoms which need to be removed as a first step when `pdb2gmx` terminates chains using the generated termini. An example is OXT, the name of one of the oxygens of the carboxylate C-terminus in Protein Data Bank entries.

findmissingpars. As a simple sanity check of the ported force field files, the presence of all required interaction parameters can be tested. After reading the previously converted force field data, the `findmissingpars` subcommand generates angle and proper dihedral interactions for all residue topologies, using the method described above, which also accounts for chaining in peptides, oligonucleotides, etc. The first check ensures that the declared atom types are actually present in the parameter set for each atom. Afterwards, interaction parameters are looked up based on the atom types. Missing parameters are reported.

As described above, in special cases, dummy parameters are written to a force field include file (`ffmissingdihedrals.itp`) to mitigate the problem of seemingly missing proper and improper dihedral types due to differences between the two MD engines. Because the `findmissingpars` subcommand and all the following subcommands work on GROMACS-formatted force field files, it is possible to reuse the same algorithms for other force fields, as well.

genhdb. Information for adding missing hydrogens to the structure by the `pdb2gmx` command is stored in `*.hdb` files in the force field directory. Employing the knowledge of the actual values of interaction parameters, the minimum energy geometry around each atom can be heuristically guessed. Based on this guess, the most suitable hydrogen atom addition rule is automatically selected, and corresponding construction entries are written.

compare. As a first step in validating the new force field port, two GROMACS-formatted force field directories can be compared using the `compare` subcommand. Differences in interaction parameters and residue topologies are listed.

rtp2itp. The `rtp2itp` subcommand can produce a `[moleculetype]` directive of a standalone residue topology from the force field directory, to be included in a topology.

Validation Procedures and Results

In CHARMM, a single-point energy is computed simply by specifying no periodic boundary conditions, infinite cutoffs (in practice specified by setting some very large value of the nonbonded cutoffs such as 999 Å) and invoking the `ENERgy` command. In GROMACS, non-periodic systems are no longer supported by the Verlet integration scheme, so the coordinates are centered in a very large cubic box with an edge length of 300 nm and nonbonded cutoffs are set to 100 nm to capture the entire molecule and approximate the more flexible behavior of CHARMM. No constraints are applied in either software to fully evaluate all bonded parameters, including those of bonds containing hydrogen atoms.

Two condensed-phase systems are also a part of the test suite, one of pure CHARMM-modified TIP3P water and another that is solution of 5 ion pairs of NaCl in water. Both systems are 40-Å cubic boxes with 1991 water molecules each. Energies of these systems were computed using periodic boundary conditions and finite cutoffs (14-Å neighbor list, smooth particle mesh Ewald

with a 12-Å real-space Coulomb cutoff, and a LJ force switch over 10-12 Å). It has previously been demonstrated that the nonbonded schemes in CHARMM and GROMACS are functionally equivalent, despite some small differences like the value of Coulomb's constant (which is slightly different in nearly every major MD simulation package) and other algorithmic differences [1]. Results of the validation energy evaluations are shown in Tables S1-S9. The component terms of the potential energy (bonded interactions, electrostatic and Van der Waals contributions) are tabulated in an additional Microsoft® Excel® workbook, being the second part of the Supporting Information.

Table S1. Potential energies (kcal mol^{-1}) for Ala-Xaa-Ala tripeptides generated with ionized termini. ALAD (alanine dipeptide) is a standalone residue with capped termini.

Residue	CHARMM	GROMACS
ALA	21.764	21.764
ALAD	-0.318	-0.317
ARG	-205.193	-205.194
ASN	-39.893	-39.891
ASP	10.403	10.405
CYS	32.493	32.493
GLN	-10.999	-10.998
GLU	23.634	23.634
GLY	12.460	12.462
HSD	28.941	28.940
HSE	-4.168	-4.168
HSP	24.871	24.871
ILE	31.513	31.510
LEU	20.067	20.071
LYS	26.156	26.157
MET	25.895	25.897
PHE	41.026	41.026
PRO	56.652	56.646

SER	38.494	38.491
THR	17.953	17.955
TRP	52.972	52.972
TYR	24.103	24.103
VAL	26.750	26.749

Table S2. Potential energies (kcal mol^{-1}) for $(Xaa)_3$ tripeptides generated with neutral termini.

Residue	CHARMM	GROMACS
ALA	78.486	78.484
ARG	-432.915	-432.904
ASN	-107.173	-107.169
ASP	103.436	103.437
CYS	103.088	103.086
GLN	-33.361	-33.360
GLU	162.295	162.293
GLY	64.006	64.006
HSD	65.822	65.821
HSE	11.944	11.946
HSP	312.808	312.801
ILE	117.900	117.902
LEU	67.907	67.907
LYS	245.042	245.036
MET	96.732	96.730
PHE	122.726	122.726
SER	125.400	125.391
THR	67.469	67.469
TRP	185.474	185.469
TYR	85.778	85.774
VAL	102.215	102.217

Table S3. Potential energies (kcal mol^{-1}) for DNA trinucleotides.

Residue	CHARMM	GROMACS
ADE	46.239	46.245
CYT	-193.935	-193.924

GUA	-273.532	-273.521
THY	-181.947	-181.939

Table S4. Potential energies (kcal mol^{-1}) for RNA trinucleotides.

Residue	CHARMM	GROMACS
ADE	217.987	217.996
CYT	-14.163	-14.161
GUA	-100.101	-100.092
URA	-27.494	-27.498

Table S5. Potential energies (kcal mol^{-1}) for selected phospholipids.

Residue	CHARMM	GROMACS
DPPC	7.788	7.791
POPA	-40.711	-40.708
POPC	11.722	11.723
POPE	0.528	0.538
POPS	-18.818	-18.816

Table S6. Potential energies (kcal mol^{-1}) for selected monosaccharides.

Residue	Molecule	CHARMM	GROMACS
AGLC	α-D-glucopyranose	105.421	105.419
BGLC	β-D-glucopyranose	111.845	111.845
AGAL	α-D-galactopyranose	106.435	106.433
BGAL	β-D-galactopyranose	111.387	111.386

Table S7. Potential energies (kcal mol^{-1}) for selected CGenFF model compounds.

Residue	Molecule	CHARMM	GROMACS
ACET	Acetate	-47.833	-47.832
BENZ	Benzene	13.162	13.163
BUTA	Butane	3.997	3.997
DMDS	Dimethyldisulfide	-2.560	-2.560
ETAM	Ethanolamine	32.627	32.626
IMIA	Imidazole (δ protonated)	-4.082	-4.082
MBOA	Methylbenzoate	37.370	37.370
MP_0	Methylphosphate (neutral)	-105.335	-105.331

OXAZ	Oxazole	5.011	5.013
UREA	Urea	-157.248	-157.244

Table S8. Potential energies and individual components (kcal mol^{-1}) for the pure water system.

MD engine	Total	Bonded[a]	LJ	Elec
CHARMM	-23018.242	1195.908	2962.465	-27176.057
GROMACS	-23021.511	1195.901	2958.341	-27175.673

[a] Sum of both bond stretching and angle bending energies.

Table S9. Potential energies and individual components (kcal mol^{-1}) for the aqueous NaCl system.

MD engine	Total	Bonded[a]	LJ	Elec
CHARMM	-23660.319	1217.175	2982.182	-27859.677
GROMACS	-23663.086	1217.173	2978.011	-27858.815

[a] Sum of both bond stretching and angle bending energies.

Demonstration of the Correctness of the Ported Parameters with Molecular Dynamics Simulations

In addition to proving the equivalence of the ported force field parameter set by calculating conformational energy terms, we have also performed actual molecular dynamics simulations on two systems using different molecular dynamics software. Input files including coordinates, topologies as well as scripts for equilibrating the system and executing the production runs were created with CHARMM-GUI[2] for CHARMM, GROMACS, and OpenMM. Simulations were also performed using the GROMACS input files made by CHARMM-GUI, but with a topology generated by the `pdb2gmx` utility of GROMACS, using the force field files ported by `charmm2gmx`. For equilibrating and production runs, the original scripts and procedures from CHARMM-GUI were retained.

As the computational performance of the CHARMM engine is substantially lower than that of GROMACS, only a fraction of the simulation time available with the latter could be achieved in reasonable time (~1 ns in ~10 days compared to ~20 ns in ~1 day). The results using the

`charmm2gmx`-ported parameters have therefore been compared with simulations performed with OpenMM and GROMACS, using the original inputs produced by CHARMM-GUI. Input and output files for these simulations, as well as various scripts used for orchestration of the runs and analysis of the results are available on Zenodo (https://doi.org/10.5281/zenodo.7997865).

Lysozyme in water

Coordinates for hen egg-white lysozyme were downloaded from the Protein Data Bank (deposit ID 1AKI). MD input files were created with the Solution Builder utility of CHARMM-GUI. Equilibration and production run was performed with CHARMM, OpenMM, and GROMACS, the latter with force field parameters and topologies supplied by CHARMM-GUI and `charmm2gmx`.

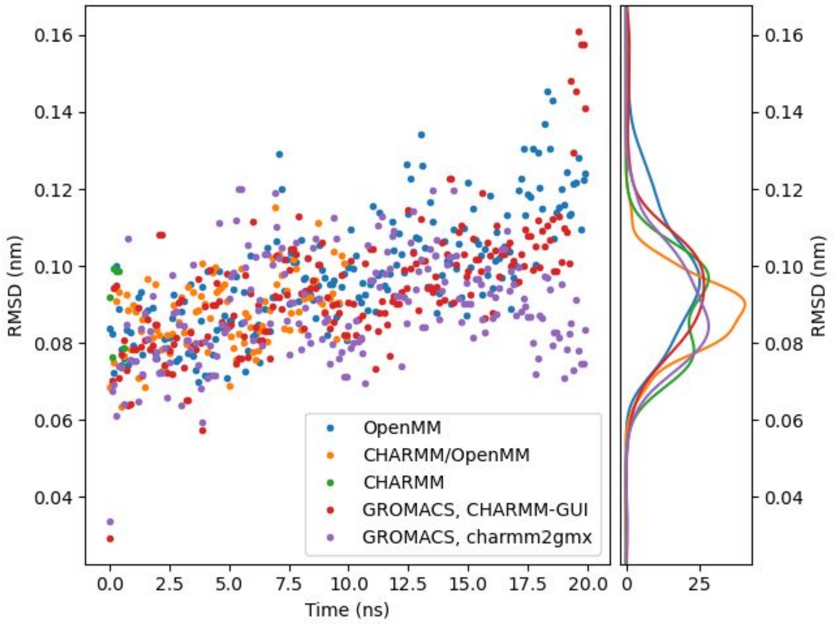

Figure S2. Root-mean-square deviation of the C_α atoms from the 1AKI crystal structure of lysozyme over time (left), and the Gaussian kernel density estimates of the time series (right), from simulations with the CHARMM36 force field using different molecular dynamics engines.

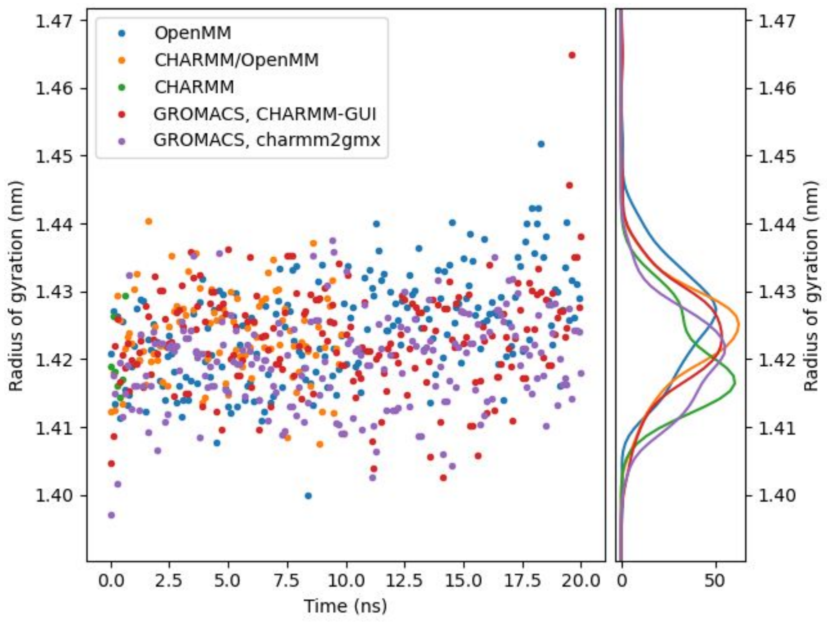

Figure S3. Time dependence (left) and Gaussian kernel density estimate (right) of the radius of gyration of lysozyme, simulated under different molecular dynamics engines.

Figures S2 and S3 show the root-mean-square deviation of C_α atoms and the radius of gyration of the whole protein, respectively. Both data series follow similar time-dependence in all cases, regardless of the molecular simulation engine or the force field conversion used.

Phospholipid Bilayer of Dipalmitoylphosphatidylcholine

A symmetric patch of 128 molecules (64 in each leaflet) of 1,2-dipalmitoyl-*sn*-glycero-3-phosphocholine (DPPC) was set up in CHARMM-GUI and simulated under CHARMM, OpenMM, and GROMACS (using both CHARMM-GUI and `charmm2gmx` parameters). Commonly analyzed quantities, such as the average area per lipid (APL), membrane thickness (distance between the opposing headgroup layers), the average angle adopted by the lipid tails with the membrane normal (z), and the transverse electron density have been calculated and are shown in Figures S4-S7. In all cases, results of simulations with OpenMM and GROMACS (the latter both with CHARMM-GUI and `charmm2gmx` parameters) agree well. Not surprisingly

CHARMM is an outlier here, producing broad distributions of all studied quantities, due to the significantly shorter trajectories we were able to produce with it (8 vs. 100 ns).

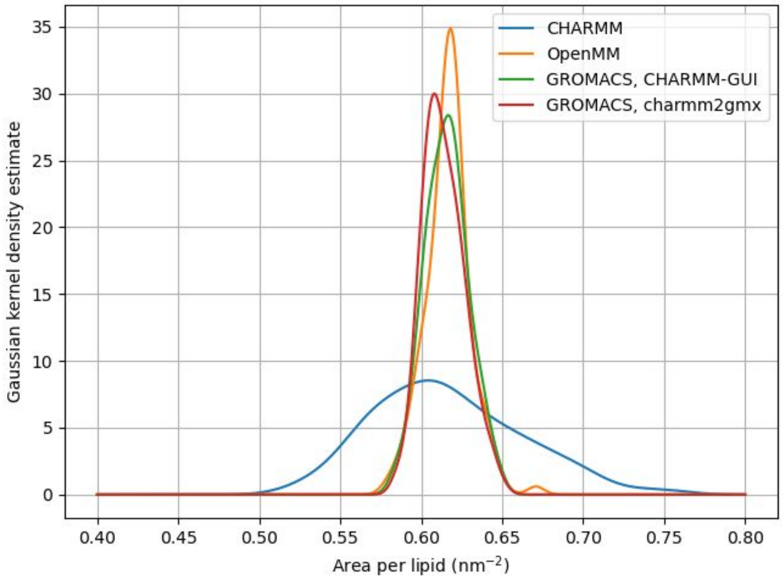

Figure S4. Estimated distribution of the average lipid footprint area in the simulation of a DPPC membrane patch under different MD engines.

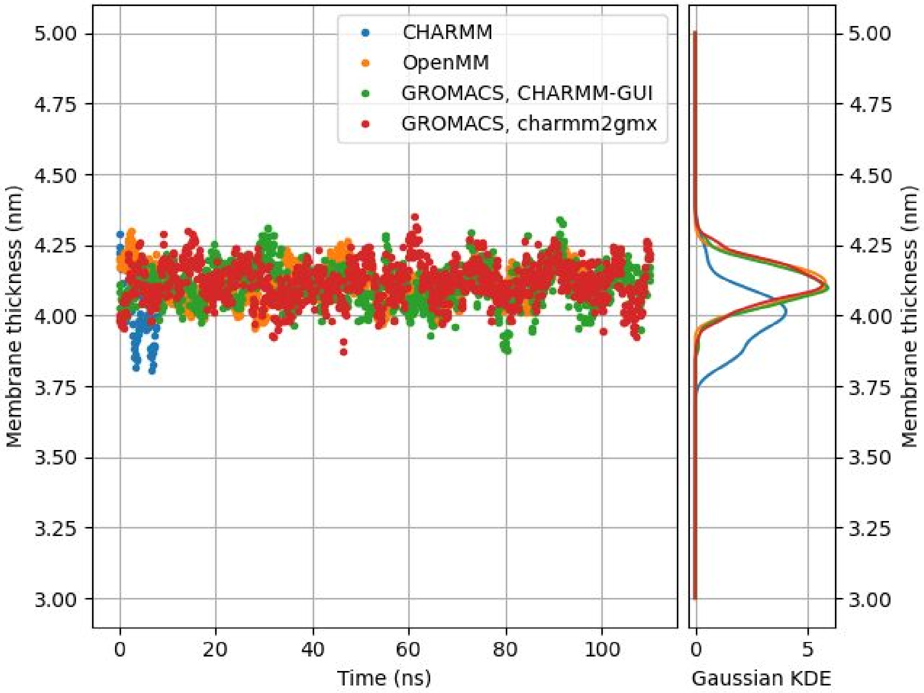

Figure S5. Time evolution of the membrane thickness (distance of the opposing headgroup layers) in the simulation of a DPPC membrane patch using different molecular dynamics engines.

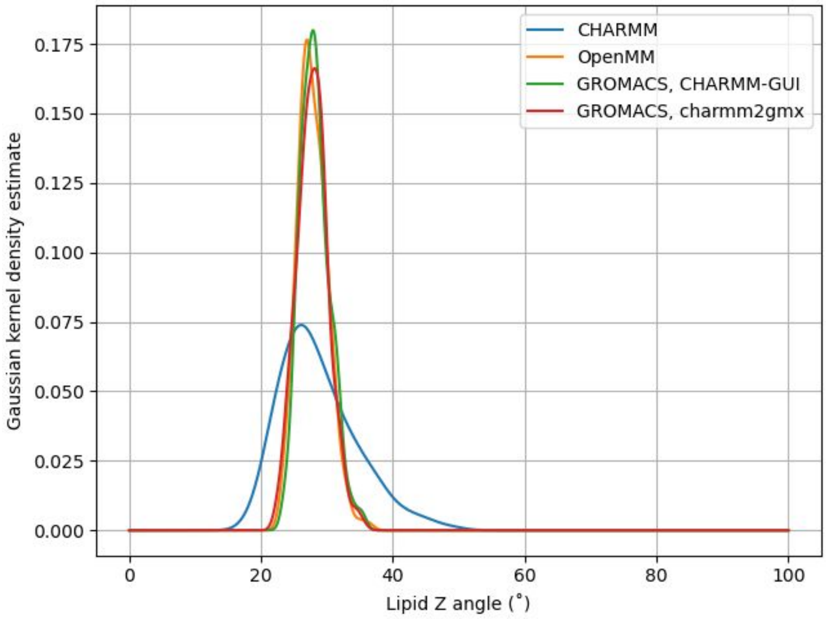

Figure S6. Estimated distribution of the average angle of lipid carbon chains with the membrane normal (z axis) studied under different MD engines.

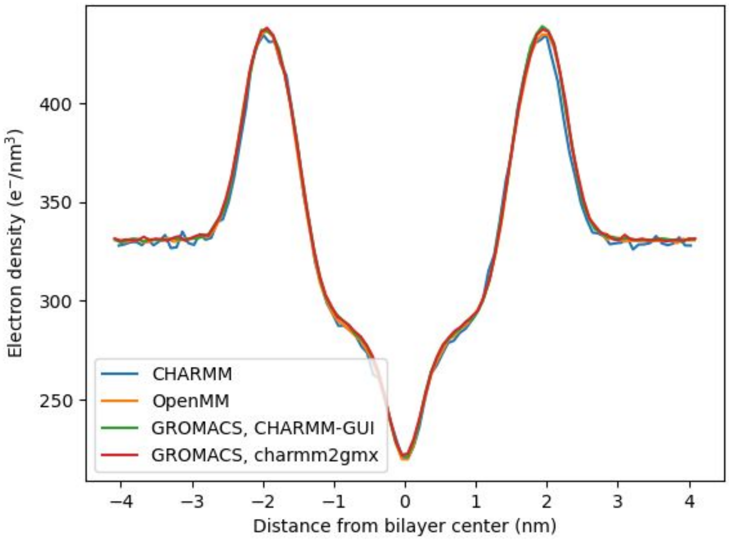

Figure S7. Electron density distribution along the membrane normal (z axis) of a DPPC membrane patch, simulated with different MD engines.

We feel the need to emphasize, though, that these MD simulations are no more effective in proving the correctness of the parameters ported with our software than the single-point

conformational energy calculations described in the main text of the article. In fact, algorithmic differences in the implementation of the respective molecular simulation codes might also introduce differences on their own, or even mask slight discrepancies or mistakes by e.g. enforcing a stable temperature and pressure on the system. That said, we find the results of the above simulations encouraging that force field ports produced by our conversion software will facilitate the easier use of the versatile CHARMM force field in a powerful and high-performance simulation software package.

Sample charmm2gmx input file for the conversion of the 2022 release of the CHARMM force field

```
; CHARMM2GMX input file
;
; FILE FORMAT
; ===========
;
; - characters including and after a semicolon (';') are comments
; - sections are denoted with [ sectionname ] and correspond to molecule
;   classes (proteins, nucleic acids etc.)
; - other lines must be empty or have the format 'keyword: value'.
;
; Top-level keywords
; ------------------
; the following keywords, given before any section header, control the
; working of the CHARMM2GMX script:
; - toppardir: directory containing an original CHARMM force field in
;    CHARMM format
; - outputdir: GROMACS-formatted FF directory to write results to.
; - bibtexfile: a BibTeX file to fetch citations from. Currently only
;    @article and @phdthesis entries are understood, in the format
;    written by the Better BibTex extension of Zotero.
; - charmmversion: CHARMM version (e.g. 36) to state in the output files
; - cgenffversion: CGenFF version (e.g. 4.6) to state in the output files
;
; Section-level keywords
; ----------------------
; 1. input file specifiers
;   prm: <filename>
;        indicates a parameter file to parse
;   rtf: <filename>
;        indicates a residue topology file to parse
;   str: <filename>
;        indicates a stream file to parse. Stream parsing is limited: READ,
;        RETURN and END commands are mostly understood, but probably nothing
;        else works.
; Each of the above keywords may be prefixed with + or -, which controls
; the handling of repeated/redefined parameters. If not prefixed,
; redefinition of interaction parameters with the same atom types but
; different parameter values is a fatal error. With '+', new parameter
; values override previously read ones. With '-', first read values take
; precedence.
; A warning message is always printed whenever such a redefinition occurs.
; Note that this only works for interaction parameters and not for residues ; or
patches. Multiple definitions of those can be resolved by
; `discardresidues` and `overrideresidues`, see below. When a file contains
; more than one definition of a residue topology, the latest one is
; retained.
;
; 2. the following keywords modify the most recently defined (via prm, rtf,
;  str keys) input file:
;    cite[description]: citekey
;    cite: citekey
;    cite[description]: "Formatted citation"
;    cite: "Formatted citation"
;         attach citations to the most recently read file. A description can
;         be given (e.g. [pyranose monosaccharides]).
;         Generally, the citation should have a corresponding entry in the
;         BibTeX file. Otherwise, e.g. when the parameters come from
;         personal communication, instead of the citation key, the text can
;         be given between double quotes, e.g. "Submitted by Sam Tonddast-
;         Navaei of U. Cincinnati. Advised by Russell Devane".
```

```
;    renameresidues: <oldname1> -> <newname1>  <oldname2> -> <newname2> ...
;          a residue, named <oldname> and read from the recent file will be
;          renamed to <newname>
;    copyresidues: <oldname1> -> <newname1>  <oldname2> -> <newname2> ...
;          a residue, named <oldname> and read from the recent file will be
;          renamed to <newname>. The original will also be retained, in
;          contrast to the `renameresidues` directive.
;    discardresidues: <resn1> <resn2> ...
;          discard (forget) residues read from the recent input file.
;          Typically used when these residues were already read from a
;          previous file. Similar to the '-' prefix before a .str or a .rtf
;          file, but gives more fine-grained control
;    overrideresidues: <resn1> <resn2> ...
;          if already read from previous files, override them with the
;          topologies read from this file. Similar effect as the "+" prefix
;          before a .str or a .rtf file, but allows more fine-grained
;          control.
;    renameatomtype: <oldname1> -> <newname1> <oldname2> -> <newname2> ...
;          similar as in renameresidues, but atom types are renamed in
;          parameter and topology files.
;    renameatoms[residuename]: <old1> -> <new1> <old2> -> <new2> ...
;           rename an atom in a residue read from the previous file.
; 3. although the following are given under sections, they are executed
;   after all files (in all sections) have been loaded.
;    patch: <base> + <pres> = <patchedname>
;          apply a patch residue <pres> onto a base residue <base> and name
;          the resulting topology <patchedname>. Both the base and patch
;          residues are looked for among all residue topologies read, not
;          just from the current file.
;    nter[baseresidue]: pres1 pres2 ...
;          convert patch residues <pres1>, <pres2> etc. to left terminals
;          (N for proteins, 5' for nucleic acids etc.). Use <baseresidue> as
;          a template residue for sorting things out.
;    cter[baseresidue]: pres1 pres2 ...
;          convert patch residues <pres1>, <pres2> etc. to right terminals
;          (C for proteins, 3' for nucleic acids etc.). Use <baseresidue> as
;          a template for sorting things out.
;    cter_delete: atomname1 atomname2 ...
;    nter_delete: atomname1 atomname2 ...
;          When creating termini database entries (in *.c.tdb or *.n.tdb
;          files), add additional [ delete ] clauses for the named atoms.
;          This can be useful for automatically removing atom OXT frequently
;          found in protein structures obtained from the Protein DataBank.
;    arn: residuename gromacsterminology charmmterminology
;          construct a line in the corresponding atom renaming database with
;          the above information, e.g. "* H HN"
;
; CHARMM2GMX execution sequence:
;    - this file is parsed
;    - all files are loaded, in the same order as found in this file
;    - after loading each file, "renameresidues", "copyresidues",
;      "renameatomtype", "renameresidues" instructions are executed on the
;      data loaded from that file.
;    - after all files have been loaded, duplicate residue topologies (and
;      patches) are sorted out using the "overrideresidues" or
;      "discardresidues" clauses.
;    - some patches are converted to stand-alone residues using the "patch"
;      commands.
;    - atom renaming database entries are constructed in each molecule class
;    - finally, termini entries are constructed in each molecule class
;      (defined by the sections), based on the instructions "cter", "nter",
;      "cter_delete", "nter_delete"
;
charmmversion: 36
cgenffversion: 4.6
toppardir: toppar_c36_jul22  ; change this to where the CHARMM files are located
outputdir: charmm36-nov2022.ff  ; the output directory
bibtexfile: citations.bib
```

```
[ solvent ]
; the main water and ions file. We need the '+' because NBFIXes (e.g.
; between SOD and CLA) are overridden later down in the file
+str: toppar_water_ions.str
cite[TIP3P water]: jorgensenComparisonSimplePotential1983
cite[Ions by Roux et. al.]: beglovFiniteRepresentationInfinite1994
cite[Zinc ion]: stoteZincBindingProteins1995
copyresidues: TIP3 -> HOH
renameatoms[HOH]: OH2 -> OW H1 -> HW1 H2 -> HW2
; the follwing is also found in
; metals/CHARMM_METAL/top_all22_prot_metals.inp
overrideresidues: CAL CES CLA MG POT SOD TP3M ZN2
; the following is also found in silicates/toppar/top_silicates.inp and
; metals/CHARMM_METAL/top_all22_prot_metals.inp
overrideresidues: TIP3
; copies of some ions to conform to legacy code
copyresidues: LIT -> LI SOD -> NA POT -> K CES -> CS CLA -> CL ZN2 -> ZN
; CAL -> CA residue renaming is not done: CA exists in CGenFF (cholic acid)
renameatoms[LI]: LIT -> LI
renameatoms[NA]: SOD -> NA
renameatoms[K]: POT -> K
renameatoms[CL]: CLA -> CL
str: stream/misc/toppar_ions_won.str
cite[Ions by Youngdo Won]: wonForceFieldMonovalent2012
[ ethers ]
prm: par_all35_ethers.prm
cite: vorobyovAdditiveClassicalDrude2007
cite: leeMolecularDynamicsStudies2008
rtf: top_all35_ethers.rtf
cite: vorobyovAdditiveClassicalDrude2007
cite: leeMolecularDynamicsStudies2008
; the following is also found in top_all36_cgenff.rtf
overrideresidues:  BUTA DECA DME ETHA HEXA IBUT THP THF PENT
overrideresidues:  PEGM HYD1 MET1 HYD2 MET2 NEOP
; Create termini for poly(ethylene-glycol)
nter[PEGM]: HYD1
nter[PEGM]: MET1
cter[PEGM]: HYD2
cter[PEGM]: MET2
[ carb ]
prm: par_all36_carb.prm
cite[pyranose monosaccharides]: guvenchAdditiveEmpiricalForce2008
cite[linear sugars, sugar alcohols, and inositol]: hatcherCHARMMAdditiveAllAtom2009
cite[hexopyranose glycosidic linkages]: guvenchCHARMMAdditiveAllAtom2009
cite[furanose monosaccharides]: hatcherCHARMMAdditiveAllAtom2009a
cite[glycosidic linkages involving furanoses]: ramanCHARMMAdditiveAllAtom2010
cite[carbohydrate derivatives and glycosidic linkages for glycoproteins]:
guvenchCHARMMAdditiveAllAtom2011
cite[O-glycan linkages]: mallajosyulaInfluenceSolventIntramolecular2011
cite[Phosphates and sulfates]: mallajosyulaCHARMMAdditiveAllAtom2012
rtf: top_all36_carb.rtf
cite[pyranose monosaccharides]: guvenchAdditiveEmpiricalForce2008
cite[linear sugars, sugar alcohols, and inositol]: hatcherCHARMMAdditiveAllAtom2009
cite[hexopyranose glycosidic linkages]: guvenchCHARMMAdditiveAllAtom2009
cite[furanose monosaccharides]: hatcherCHARMMAdditiveAllAtom2009a
cite[glycosidic linkages involving furanoses]: ramanCHARMMAdditiveAllAtom2010
cite[carbohydrate derivatives and glycosidic linkages for glycoproteins]:
guvenchCHARMMAdditiveAllAtom2011
cite[O-glycan linkages]: mallajosyulaInfluenceSolventIntramolecular2011
cite[Phosphates and sulfates]: mallajosyulaCHARMMAdditiveAllAtom2012
str: stream/carb/toppar_all36_carb_glycolipid.str
cite: kernLipidLinkedOligosaccharidesMembranes2014
str: stream/carb/toppar_all36_carb_glycopeptide.str
str: stream/carb/toppar_all36_carb_imlab.str
str: stream/carb/toppar_all36_carb_lignin.str
cite: vermaasAutomatedTransformationLignin2019
cite: v.vermaasSystematicParameterizationLignin2019
[ lipid ]
```

```
prm: par_all36_lipid.prm
cite: klaudaUpdateCHARMMAllAtom2010
cite: klaudaImprovingCHARMMForce2012
rtf: top_all36_lipid.rtf
cite: klaudaUpdateCHARMMAllAtom2010
cite: klaudaImprovingCHARMMForce2012
str: stream/lipid/toppar_all36_lipid_bacterial.str
cite[Branched lipids]: limLipidChainBranching2011
cite[Cyclic-replacement of double bond]: panditMembraneModelsColi2012
copyresidues: OYPE -> OSPE PYPG -> PSPG
renameresidues: PHPC -> PhPC
str: stream/lipid/toppar_all36_lipid_cardiolipin.str
copyresidues: TYCL1 -> TXCL1 TYCL2 -> TXCL2
str: stream/lipid/toppar_all36_lipid_cholesterol.str
cite[Original model]: pitmanMolecularLevelOrganizationSaturated2004
cite[Updated model]: limUpdateCholesterolForce2012
str: stream/lipid/toppar_all36_lipid_detergent.str
copyresidues: C7DHPC -> DHPC FOS12 -> DPC FOS14 -> TPC
str: stream/lipid/toppar_all36_lipid_ether.str
cite: leonardParameterizationCHARMMAllAtom2018
str: stream/lipid/toppar_all36_lipid_inositol.str
str: stream/lipid/toppar_all36_lipid_lps.str
str: stream/lipid/toppar_all36_lipid_miscellaneous.str
copyresidues: DYPA -> DXPA DYPG -> DXPG DYPS -> DXPS
str: stream/lipid/toppar_all36_lipid_prot.str
str: stream/lipid/toppar_all36_lipid_sphingo.str
cite[PSM and SSM lipids]: venableCHARMMAllAtomAdditive2014
cite[Ceramide]: "Submitted by Sam Tonddast-Navaei of U. Cincinnati. Advised by
Russell Devane"
str: stream/lipid/toppar_all36_lipid_yeast.str
[ na ]
prm: par_all36_na.prm
cite: foloppeAllatomEmpiricalForce2000
cite: mackerellAllatomEmpiricalForce2000
cite: denningImpactHydroxylSampling2011
cite: hartOptimizationCHARMMAdditive2012
rtf: top_all36_na.rtf
cite: foloppeAllatomEmpiricalForce2000
cite: mackerellAllatomEmpiricalForce2000
cite: denningImpactHydroxylSampling2011
cite: hartOptimizationCHARMMAdditive2012
nter[CYT]: 5TER 5MET 5PHO 5POM
cter[CYT]: 3TER
+str: stream/na/toppar_all36_na_modifications.str
; the following is also found in toppar_all36_cgenff.rtf
overrideresidues:  ABNS B5NP B5SP BPET BPNP BPSP CNAD CNCY CNGU CNT1 CNT2 CNT3 CNTH
CPES CSAD CSCY CSGU CSTH THF2 THNI THNP TPHC PHA DFB DFT
; create deoxy residues
patch: CYT + DEOX = DC
patch: ADE + DEOX = DA
patch: GUA + DEOX = DG
patch: THY + DEOX = DT
patch: URA + DEOX = DU
; Note: 3'- and 5'-terminal deoxy residues are superfluous, can be done with
; pdb2gmx termini patching
;; create 5'-terminal deoxy residues
;patch: DC + 5TER + DC = DC5
;patch: DA + 5TER + DA = DA5
;patch: DG + 5TER + DG = DG5
;patch: DT + 5TER + DT = DT5
;patch: DU + 5TER + DU = DU5
;; create 3'-terminal deoxy residues
;patch: DC + 3TER = DC3
;patch: DA + 3TER = DA3
;patch: DG + 3TER = DG3
;patch: DT + 3TER = DT3
;patch: DU + 3TER = DU3
patch: DC + 5MC2 = D5MC
```

```
cite[Carbocyclic sugar parameters]:
wangUseOligodeoxyribonucleotidesConformationally2000
cite[Locked nucleic acids]: hartonoModelingPKShift2018
cite[Peptide nucleic acids]: jasinskiImprovedForceFields2018
-str: stream/na/toppar_all36_na_nad_ppi.str
; also present in top_all36_cgenff.rtf
overrideresidues:  ADP AMP ATP  5DP NAD NADH NADP NDPH NIC NICH PPI1 PPI2
cite[NAD+, NADH, ADP, ATP, PPI and related analogs]:
pavelitesMolecularMechanicsForce1997
;-str: stream/na/toppar_all36_na_rna_modified.str
;cite[Modified ribonucleic acids]: xuAdditiveCHARMMForce2016
[ aminoacids ]
prm: par_all36m_prot.prm
cite: mackerellAllAtomEmpiricalPotential1998
cite: mackerellImprovedTreatmentProtein2004
cite: bestOptimizationAdditiveCHARMM2012
cite: huangCHARMM36mImprovedForce2016
; we need the '+' in the next line: atomtype O in the prm file has mass 15.999, while
in the rtf 15.9994
+rtf: top_all36_prot.rtf
cite: mackerellAllAtomEmpiricalPotential1998
cite: mackerellImprovedTreatmentProtein2004
cite: bestOptimizationAdditiveCHARMM2012
cite: huangCHARMM36mImprovedForce2016
; the following are also found in metals/CHARMM_METAL/top_all22_prot_metals.inp
overrideresidues: CT2 DISU
renameresidues: GLYP -> GLY-NH3+
renameresidues: PROP -> PRO-NH2+
renameresidues: NGNE -> GLY-NH2
renameresidues: PCTE -> COOH
renameresidues: CTER -> COO-
renameresidues: NTER -> NH3+
renameresidues: NNEU -> NH2
renameatoms[NH2]: HT1 -> H1 HT2 -> H2
renameatoms[NH3+]: HT1 -> H1 HT2 -> H2 HT3 -> H3
renameatoms[GLY-NH3+]: HT1 -> H1 HT2 -> H2 HT3 -> H3
patch: ASP + ASPP = ASPP
patch: GLU + GLUP = GLUP
patch: LYS + LSN = LSN
patch: CYS + CYSD = CYN
patch: CYS + DISU = CYS2
patch: SER + SERD = SERD
nter[GLY]: GLY-NH3+
nter[ALA]: NH3+
nter[PRO]: PRO-NH2+
nter[GLY]: GLY-NH2
nter[ALA]: NH2
cter[GLY]: COO- COOH CT2
; CT1 is methylated C-terminus. top_all36_prot.rtf: "don't use with Gly or Pro" =>
use ALA as base.
cter[ALA]: CT1
cter_delete: OXT
str: stream/prot/toppar_all36_prot_heme.str
renameresidues: CO2 -> CO2M
; traditionally, MES2 is available under the name MESM in the GROMACS port. There are
no MES2 residue in the CHARMM
; toppar files, so this renaming is deprecated.
copyresidues: MES2 -> MESM
str: stream/prot/toppar_all36_prot_c36m_d_aminoacids.str
; the following is also present in stream/prot/toppar_all36_prot_modify_res.str
overrideresidues: DILE
; the following is also present in top_all35_ethers.rtf
overrideresidues: DMET
str: stream/prot/toppar_all36_prot_modify_res.str
overrideresidues: HIC HICP
; also appears in metals/CHARMM_METAL/top_all22_prot_metals.inp
overrideresidues: AGM
patch: LYS + KAC = KAC
```

```
patch: HSE + MHSE = MHSE
patch: HSD + MHSD = MHSD
patch: HSP + HSPM = HSPM
str: stream/prot/toppar_all36_prot_retinol.str
; the following is also found in top_all36_cgenff.rtf
overrideresidues:  13DB 13DP BTE2 CROT DMB1 DMP1 DMP2 FRET MECH PACP PRAC PRAL PROL
RTAC RTAL RTOL SCH1 SCH2 SCH3 TMCH
str: stream/prot/toppar_all36_prot_arg0.str
patch: ARG + RN1 = ARGN1
patch: ARG + RN2 = ARGN2
patch: ARG + RN3 = ARGN3
patch: ARG + RN2 = ARGN
cite[Neutral arginine]: liArginineChargedMembrane2008
str: stream/prot/toppar_all36_prot_fluoro_alkanes.str
discardresidues: TFE TFET FETH DFET
cite[Fluoro alkanes]: chenCombinedInitioEmpirical2002
str: stream/prot/toppar_all36_prot_na_combined.str
cite[Phosphotyrosine]:  fengStructuralCharacterizationPhosphotyrosine1996
patch: SER + SP1 = SP1
patch: SER + SP2 = SP2
patch: THR + THP1 = THP1
patch: THR + THPB = THP2
patch: TYR + TP1 = TP1
patch: TYR + TP2 = TP2
patch: TYR + TP1A = TP1A
patch: TYR + TP2A = TP2A
patch: TYR + TMP1 = TMP1
patch: TYR + TMP2 = TMP2
patch: TYR + TDF1 = TDF1
patch: TYR + TDF2 = TDF2
patch: ADE + SAHC = SAC
patch: ADE + PSAM = PSAM
str: stream/prot/toppar_all36_prot_model.str
; the following residues are also found in
stream/prot/toppar_all36_prot_modify_res.str
overrideresidues: CGU CGUP CME
arn: * H HN
[ silicates ]
rtf: silicates/toppar/top_silicates.inp
renameresidues: SIM1 -> SiM1 SIM2 -> SiM2 SIO4 -> SiO4
renameatomtype: AL -> ALSI
-prm: silicates/toppar/par_silicates.inp
renameatomtype: AL -> ALSI
cite: lopesDevelopmentEmpiricalForce2006
[ metals ]
; the following line needs '-' to avoid overriding CHARMM36 interaction parameters
-rtf: metals/CHARMM_METAL/top_all22_prot_metals.inp
discardresidues: ALA ARG ASN ASP CYS GLN GLU GLY HSD HSE HSP ILE LEU LYS MET PHE PRO
SER THR TRP TYR VAL ALAD NTER GLYP
discardresidues: PROP ACE ACP CT1 CT3 ASPP GLUP LSN LINK HS2 LIG1 ACE NME
cite: heinzAccurateSimulationSurfaces2008
cite: heinzNatureMolecularInteractions2009
cite: fengAdsorptionMechanismSingle2011
cite: coppageCrystallographicRecognitionControls2011
cite: fengInfluenceShapeNanostructured2012
-prm: metals/CHARMM_METAL/par_all22_prot_metals.inp
cite: heinzAccurateSimulationSurfaces2008
cite: heinzNatureMolecularInteractions2009
cite: fengAdsorptionMechanismSingle2011
cite: coppageCrystallographicRecognitionControls2011
cite: fengInfluenceShapeNanostructured2012
[ cgenff ]
-prm: par_all36_cgenff.prm
cite: vanommeslaegheCHARMMGeneralForce2010
cite: yuExtensionCHARMMGeneral2012
rtf: top_all36_cgenff.rtf
cite: vanommeslaegheCHARMMGeneralForce2010
cite: yuExtensionCHARMMGeneral2012
```

```
; the following residues are also found in stream/prot/toppar_all36_prot_retinol.str
;overrideresidues:
; the following residues are also found in stream/prot/toppar_all36_prot_model.str
overrideresidues: ACEH ACEM ACET BENZ C3 CO3 DEDS DMDS EAMM EBEN EIMI EIMM EIND EMS
EPHE ES1 ETO ETOH ETSH FORM GLYN GUAN IMIA IMIM INDO MAMM MEOH MES1 MESH METO MGUA
MIMI NMA OCOH PDIP PHEN PHEO PRO2 PROA PRPA
; the following residues are also found in stream/na/topar_all36_na_modifications.str
;overrideresidues:
; the following residues are also found in stream/na/toppar_all36_na_nad_ppi.str
;overrideresidues:
; the following residues are also found in
stream/prot/toppar_all36_prot_na_combined.str
overrideresidues: BDFD BDFP BMPD BMPH EP_2 IP_2 NUCL PPH1 RNUS THAO THFA THFI THMI
; the following residues are also found in top_all35_ethers.rtf
overrideresidues: CPEN
; the following residues are also found in stream/prot/toppar_all36_prot_arg0.str
overrideresidues: MGU1 MGU2
; the following is also found in stream/misc/toppar_ions_won.str: there Thulium 3+
ion.
overrideresidues: TM3P
; The following patches modify residue GABD, i.e. Gamma amino benzene glutamic acid
; CDCA amide
patch: GABD + 4FBD = 4FBD
patch: GABD + 3FBD = 3FBD
patch: GABD + 2FBD = 2FBD
patch: GABD + 4FBP = 4FBP
patch: GABD + 3FBP = 3FBP
patch: GABD + 2FBP = 2FBP
patch: GABD + 2MBD = 2MBD
patch: GABD + 3MBD = 3MBD
patch: GABD + 4MBD = 4MBD
patch: GABD + 2XBD = 2XBD
patch: GABD + 3XBD = 3XBD
patch: GABD + 4XBD = 4XBD
patch: GABD + 4XEBD = 4XEBD
patch: GABD + 3XBP = 3XBP
patch: GABD + 2AMFD = 2AMFD
patch: GABD + 3AMFD = 3AMFD
patch: GABD + 4AMFD = 4AMFD
patch: GABD + 26MD = 26MD
patch: GABD + 23MD = 23MD
patch: GABD + 24MFD = 24MFD
patch: GABD + 35MFD = 35MFD
patch: FBIB + FBIA = FBIA
patch: FBIB + FBICS = FBICS
patch: FBIB + FBID = FBID
patch: FBIB + FBIE = FBIE
patch: FBIB + FBIFR = FBIFR
patch: FBIB + FBIFS = FBIFS
patch: BAB1 + G4MP = G4MP
patch: BAB1 + G4MPAB = G4MPAB
patch: BAB1 + G4P = G4P
patch: BAB1 + G4PAB = G4PAB
patch: BAB1 + G3PR = G3PR
patch: BAB1 + G3PS = G3PS
patch: BAB1 + G3PABR = G3PABR
patch: BAB1 + G3PABS = G3PABS
patch: BAB1 + BPAB = BPAB
patch: BAB1 + BPAT = BPAT
patch: BAB1 + GBP = GBP
patch: BAB1 + 3MSB = 3MSB
patch: BAB1 + 3MPS = 3MPS
patch: BAB1 + 2MRB = 2MRB
patch: BAB1 + 2MPR = 2MPR
patch: BAB1 + 3MRB = 3MRB
patch: BAB1 + 3MPR = 3MPR
patch: BAB1 + 2MSB = 2MSB
patch: BAB1 + 2MPS = 2MPS
```

```
patch: GA + AMGA = AMGA
patch: GA + GMGA = GMGA
patch: GA + DMGA = DMGA
patch: GA + AMGT = AMGT
patch: GA + ATGM = ATGM
patch: GA + ABGA = ABGA
patch: GA + GBGA = GBGA
patch: GA + DBGA = DBGA
patch: GA + AL = GA_AL   ; not to mix up with aluminum
patch: GA + ALME = ALME
patch: GA + 3A2MPD = 3A2MPD
patch: GA + 3A2MPP = 3A2MPP
patch: GA + 3A4MPD = 3A4MPD
patch: GA + 3A5MPD = 3A5MPD
patch: GA + 3A6MPD = 3A6MPD
patch: GA + 4A2MPD = 4A2MPD
patch: GA + 4A2MPP = 4A2MPP
patch: GA + 2APD = 2APD
patch: GA + 2APP = 2APP
patch: GA + 2AFP = 2AFP
patch: GA + 2AFD = 2AFD
patch: GA + 3APD = 3APD
patch: GA + 3AFP = 3AFP
patch: GA + 3AFD = 3AFD
patch: GA + 3APP = 3APP
patch: GA + 4APP = 4APP
patch: GA + 4APD = 4APD
patch: GA + 4AFP = 4AFP
patch: GA + 4AFD = 4AFD
patch: GA + 3CPD = 3CPD
patch: 3APD + 3A26PD = 3A26PD  ; !!! Double patch !!!
patch: 2APD + 2A46PD = 2A46PD  ; !!! Double patch !!!
patch: 2APD + 2A3HPD = 2A3HPD  ; !!! Double patch !!!
patch: GA + 3A46PD = 3A46PD
patch: GA + 2AEPD = 2AEPD
patch: CA + GYCO = GCA
patch: DCA + GYCO = GDCA
patch: LCA + GYCO = GLCA
patch: CDCA + GYCO = GCDCA
patch: UDCA + GYCO = GUDCA
[ noble ]
;-str: stream/misc/toppar_dum_noble_gases.str
;discardresidues: DUM
```

Charmm2gmx input file modifications to convert the lipid LJ-PME variant of the 2022 release of CHARMM

```
...
charmmversion: 36
cgenffversion: 4.6
toppardir: toppar_c36_jul22
outputdir: charmm36_ljpme-nov2022.ff
bibtexfile: citations.bib
...
[ lipid ]
prm: par_all36_lipid_ljpme.prm
cite[LJ-PME lipid parameters]: yuSemiautomatedOptimizationCHARMM362021
cite: klaudaUpdateCHARMMAllAtom2010
cite: klaudaImprovingCHARMMForce2012
rtf: top_all36_lipid_ljpme.rtf
cite[LJ-PME lipid parameters]: yuSemiautomatedOptimizationCHARMM362021
cite: klaudaUpdateCHARMMAllAtom2010
cite: klaudaImprovingCHARMMForce2012
str: stream/lipid/toppar_all36_lipid_bacterial.str
```

```
cite[Branched lipids]: limLipidChainBranching2011
cite[Cyclic-replacement of double bond]: panditMembraneModelsColi2012
copyresidues: OYPE -> OSPE PYPG -> PSPG
renameresidues: PHPC -> PhPC
...
```

References

(1) Lee, J.; Cheng, X.; Swails, J. M.; Yeom, M. S.; Eastman, P. K.; Lemkul, J. A.; Wei, S.; Buckner, J.; Jeong, J. C.; Qi, Y.; Jo, S.; Pande, V. S.; Case, D. A.; Brooks, C. L. I.; MacKerell, A. D. Jr.; Klauda, J. B.; Im, W. CHARMM-GUI Input Generator for NAMD, GROMACS, AMBER, OpenMM, and CHARMM/OpenMM Simulations Using the CHARMM36 Additive Force Field. *J. Chem. Theory Comput.* **2016**, *12* (1), 405–413. https://doi.org/10.1021/acs.jctc.5b00935.

(2) Jo, S.; Kim, T.; Iyer, V. G.; Im, W. CHARMM-GUI: A Web-Based Graphical User Interface for CHARMM. *Journal of Computational Chemistry* **2008**, *29* (11), 1859–1865. https://doi.org/10.1002/jcc.20945.
